# Differential effects of body mass index on domain-specific cognitive outcomes after stroke

**DOI:** 10.1038/s41598-021-93714-7

**Published:** 2021-07-08

**Authors:** Minwoo Lee, Mi Sun Oh, San Jung, Ju-Hun Lee, Chul-Ho Kim, Min Uk Jang, Young Eun Kim, Hee-Joon Bae, Jaeseol Park, Yeonwook Kang, Byung-Chul Lee, Jae-Sung Lim, Kyung-Ho Yu

**Affiliations:** 1 Department of Neurology, Hallym Neurological Institute, Hallym University Sacred Heart Hospital, 896, Anyang-City, 430-070 South Korea; 2 Department of Neurology, Kangnam Sacred Heart Hospital, Seoul, South Korea; 3 Department of Neurology, Kangdong Sacred Heart Hospital, Seoul, South Korea; 4 Department of Neurology, Chunchon Sacred Heart Hospital, Chunchon, South Korea; 5 Department of Neurology, Dongtan Sacred Heart Hospital, Hwaseong, South Korea; 6 Department of Neurology, Seoul National University Bundang Hospital, Seongnam, South Korea; 7 Department of Psychology, Hallym University, Chunchon, South Korea; 8 Department of Neurology, Asan Medical Center, University of Ulsan College of Medicine , 88, Olympic-ro 43-gil, Songpa-gu, Seoul, 05505 South Korea

**Keywords:** Dementia, Stroke

## Abstract

Although the obesity paradox is an important modifiable factor in cardiovascular diseases, little research has been conducted to determine how it affects post-stroke cognitive function. We aimed to investigate the association between body mass index (BMI) and domain-specific cognitive outcomes, focusing on the subdivision of each frontal domain function in post-ischemic stroke survivors. A total of 335 ischemic stroke patients were included in the study after completion of the Korean-Mini Mental Status Examination (K-MMSE) and the vascular cognitive impairment harmonization standards neuropsychological protocol at 3 months after stroke. Frontal lobe functions were analyzed using semantic/phonemic fluency, processing speed, and mental set shifting. Our study participants were categorized into four groups according to BMI quartiles. The z-scores of K-MMSE at 3 months differed significantly between the groups after adjustment for initial stroke severity (*p* = 0.014). Global cognitive function in stroke survivors in the Q1 (the lowest quartile) BMI group was significantly lower than those in Q2 and Q4 (the highest quartile) BMI groups (K-MMSE z-scores, Q1: − 2.10 ± 3.40 vs. Q2: 0.71 ± 1.95 and Q4: − 1.21 ± 1.65). Controlled oral word association test findings indicated that phonemic and semantic word fluency was lower in Q4 BMI group participants than in Q2 BMI group participants (*p* = 0.016 and *p* = 0.023 respectively). BMI might differentially affect cognitive domains after ischemic stroke. Although being underweight may negatively affect global cognition post-stroke, obesity could induce frontal lobe dysfunctions, specifically phonemic and semantic word fluency.

## Introduction

Obesity is a growing public health concern and a well-recognized risk factor for cardiovascular disorders and overall mortality^1^. However, an increasing body of evidence suggests that obesity has a protective effect on cardiovascular disease or increases the probability of survival in certain patients such as the elderly^2,3^. Referred to as the ‘obesity paradox’, this paradoxical phenomenon has been observed not only in cardiovascular diseases^4^, but also in cerebrovascular disorders, peripheral arterial disease^5^, diabetes mellitus^6^, malignancy^7^, and dementia^8,9^. However, it is yet controversial whether the ‘obesity paradox’ also exists in patients with cognitive disorders. While being underweight (BMI < 20 kg/m^2^) is known to be associated with an increased risk of dementia^8^, the effects of higher BMI on cognitive outcomes are inconsistent throughout the studies. Some studies have suggested that inflammation and vascular changes accompanied by obesity lead to deterioration of cognitive function^10^, while others reported that a higher BMI in middle-to-late life predicts a lower risk of developing dementia^9^.

The obesity paradox has also been observed in stroke population, however, the relationship is somewhat complex^11^. In some studies, high BMI was associated with reduced stroke severity, favorable functional outcomes, and lower mortality after stroke^12–14^. However another study showed that overweight or obesity did not confer a favorable effect while underweight could have poor outcomes after stroke^15^. Furthermore, these reports did not explore the independent association between BMI and post-stroke cognition. One Swedish study reported that BMI did not influence cognitive impairment at 20 months after stroke in a sample of 149 stroke survivors^16^. On the other hand, pre-stroke frailty was independently associated with poor post-stroke cognitive fucnction^17^. However, those studies used only the screening tools to assess cognition, which cannot be considered appropriate given the higher incidence of frontal dysfunction among stroke survivors^18^. To date, few studies have used detailed neuropsychological evaluations to determine how obesity affects post-stroke cognitive impairment^19^. It has already been reported that obesity primarily affects metabolic activity and cognitive function of the frontal lobe, but the effects of obesity on each cognitive domain in stroke patients have not yet been sufficiently studied^20,21^. This is also clinically important in properly screening patients with cognitive impairment and evaluating their treatment effectiveness.

In this context, we aimed to investigate the differential effects of BMI on domain-specific and global cognitive function at 3 months post-ischemic stroke using the Korean Vascular Cognitive Impairment Harmonization Standards-Neuropsychological Protocol (K-VCIHS-NP), a comprehensive neuropsychological test developed for post-stroke survivors. We hypothesized that BMI might have distinct roles in each neural substrate associated with each cognitive domain, as previous studies have shown that high BMI is associated with executive dysfunction and frontal lobe atrophy in a generally healthy population^20,22,23^.

## Methods

### Study participants

Participants were enrolled from the Korean Vascular Cognitive Impairment Harmonization Standards (K-VCIHS) study, which has been previously described in detail^24^. The K-VCIHS cohort enrolled patients with ischemic stroke who had been consecutively admitted to 12 university hospitals from October 2007 to August 2008. Inclusion criteria for our study comprised the following: (i) a diagnosis of acute ischemic stroke with neurological deficits persisting for ≥ 24 h, (ii) a relevant ischemic lesion observed on magnetic resonance imaging, (iii) admission within 7 days after symptom onset, and (iv) available data on admission concerning BMI and the results of K-VCIHS-NP performed at 3 months post-ischemic stroke. In total, 335 participants from the K-VCIHS cohort who fulfilled the inclusion criteria were finally analyzed. We collected clinical variables and the results of K-VCIHS-NP with the consent of the principal investigator of the K-VCIHS-NP study group and the Institutional Review Board of Hallym University Sacred Heart Hospital. BMI was calculated as a participant’s weight (kg) divided by their height (m) squared, measured on the first day of admission according to the institutional protocol with an automatic weight and height machine. Body weight and height were measured to the nearest 0.1 kg and 0.1 cm respectively using BSM-330 (InBody, South Korea). Those who were not able to stand were weighed using an electronic bed scale, SCB330-7 (SOHWA Inc, South Korea), in the supine position. The participants were then stratified into BMI quartiles^25^, namely, Q1, < 21.87 kg/m^2^; Q2, 21.88–23.87 kg/m^2^; Q3, 23.88–25.96 kg/m^2^, and; Q4, > 25.97 kg/m^2^. From the cohort registry data, we collected clinical data concerning age, sex, and stroke subtype according to the Trial of ORG 10,172 in Acute Stroke Treatment (TOAST) classification^26^. Moreover, initial stroke severity represented as the National Institute of Health Stroke Scale and vascular risk factors were also collected. The study was approved by the Hallym University Sacred Heart Hospital Institutional Review Board and conformed to the Declaration of Helsinki. The requirement for informed consent was waived the Hallym University Sacred Heart Hospital Institutional Review Board because of the retrospective nature of this study as well as the minimal risk that it posed to participants.

### Cognitive function assessment

Participants completed the Korean Mini-Mental Status Examination (K-MMSE) and a 60-min neuropsychological test as described in the K-VCIHS study protocol^27^. The K-VCIHS assesses four cognitive domains: (i) executive/activation function, using the Korean version of the controlled oral word association test (COWAT) for phonemic and semantic fluency, digit symbol coding, and trail making tests A and B; (ii) language, using the Korean-Boston naming test; (iii) visuospatial, using the Rey complex figure test (copy), and; (iv) memory, using delayed recall scores from the Seoul verbal learning test. The K-VCIHS study protocol also included the Informant Questionnaire of Cognitive Decline in the Elderly (IQCODE) for the premorbid history of cognitive dysfunctions. The specific tests and scales which comprise the K-VCIHS protocol and relevant references are described in Supplemental Table I. We obtained standardized Z-scores for each domain and for the K-MMSE after adjustment for age, sex, and educational level to directly compare groups according to BMI quartiles.

### Brain imaging

All participants underwent brain magnetic resonance imaging (MRI), performed using a 3 T whole-body MRI system. Diffusion-weighted imaging was used to obtain the apparent diffusion coefficient and assess acute cerebral infarction. The location and number of acute ischemic stroke lesions were rated and quantified. We subdivided the stroke lesions as follows: (i) cortical vs. subcortical-only, (ii) left-sided vs. right-sided, and; (iii) single vs. multiple^12^.

### Statistical analysis

In order to detect an effect η^2^p = 0.04 with 80% power in a between-subjects ANCOVA (four groups, numerator df = 3, alpha = 0.05), a priori power calculation of G*Power 3.1.9.4^28^ suggested that we would need 67 subjects in each group (n = 266). Pearson’s chi-square test and an analysis of variance (ANOVA) were used, as appropriate, to compare demographic characteristics between the groups according to BMI quartiles. For the univariate analysis, an ANOVA was used to compare the Z-scores of each cognitive domain between the groups. The Levene’s test and Kolmogorov–Smirnov tests were performed to check for equality of variances and normality of dependent variables, respectively. The prespecified covariates included in the main analysis were age, sex, education years, and initial stroke severity (NIHSS) that showed a strong association with post-stroke cognitive impairment in the previous studies^29^. An analysis of covariance (ANCOVA) was performed adjusting for the initial stroke severity (NIHSS), as age, sex, and education levels were considered in the z-score transformation process. The effect size of BMI quartiles on dependent variables in the ANCOVA was presented with partial eta squared (η^2^p). We further conducted a sensitivity analysis using cutoff points for BMI according to the Asia–Pacific classification^30^ (Underweight, < 18.5 kg/m^2^; Normal, 18.5–22.9 kg/m^2^; Overweight, 23–24.9 kg/m^2^; Obese ≥ 25 kg/m2) to determine the generalizability of our study. Two-sided *p*-values < 0.05 were considered to indicate statistical significance. All analyses were performed using IBM SPSS, version 26.

## Results

The mean age of the participants was 64.8 ± 12.4 years, and women comprised 38.9% of the study population. The mean BMI was 23.89 ± 3.10 kg/m^2^ (range, 15.82–32.93 kg/m^2^). The BMI quartile groups did not differ in terms of age, sex, years of education, previous stroke history, or pre-stroke cognitive decline. There were no significant differences in initial stroke severity, stroke etiology according to the TOAST classification, or the number and location of ischemic lesions. However, the prevalence of hypertension was higher in the Q4 BMI group (the highest) than in the Q1 BMI group (the lowest). The prevalence of other vascular risk factors did not differ significantly between the groups (Table 1).

**Table 1:** Clinical Characteristics according to the quartiles of body mass index.

	Q 1 (< 21.87, n = 84)	Q 2 (21.88–23.87, n = 84)	Q 3 (23.88–25.96, n = 84)	Q 4 (> 25.97, n = 83)	*P* values
Age, mean ± SD	66.2 ± 14.6	65.8 ± 12.8	63.8 ± 11.1	63.3 ± 10.9	0.329
Women, %(N)	46.4(39)	42.9 (36)	27.4 (23)	39.8 (33)	0.064
**Vascular risk factors**					
Hypertension, %(N)	54.8 (46)	63.1 (53)	58.3 (49)	74.7 (62)	0.045*
Diabetes, %(N)	31.0 (26)	23.8 (20)	33.3 (28)	39.8 (33)	0.172
Dyslipidemia, %(N)	31.0 (26)	23.8 (20)	33.3 (28)	39.8 (33)	0.085
History of Stroke, %(N)	23.8 (20)	14.3 (12)	20.2 (17)	27.4 (23)	0.194
Smoking, %(N)	44.0 (37)	42.9 (36)	50.0 (42)	43.4 (36)	0.772
Atrial fibrillation, %(N)	13.1 (11)	16.7 (14)	16.7 (14)	14.5 (12)	0.896
**Education**					0.148
Illiterate, %(N)	9.5 (8)	6.0 (5)	1.2 (1)	6.0 (5)	
0–6 years, %(N)	31.0(26)	48.8(41)	38.1(32)	28.9(24)	
7–12 years, %(N)	39.2 (33)	31.0(26)	38.1(32)	47.0(39)	
13 years and more, %(N)	20.2 (17)	14.3 (12)	22.6 (19)	18.1 (15)	
Prestroke Cognitive Impairment^a^, %(N)	8.3 (7)	3.6 (3)	7.1 (6)	8.4 (7)	0.565
NIHSS score, median [IQR]	2.5[1.0;5.5]	3.0[2.0;5.0]	3.0[1.5;6.0]	3.0[1.0;5.0]	0.942
**Ischemic stroke subtype**					0.813
Large artery atherosclerosis, %(N)	39.3 (33)	34.5 (29)	42.9 (36)	47.0 (39)	
Small vessel occlusion, %(N)	26.2 (22)	33.3 (28)	25.0 (21)	26.5 (22)	
Cardioembolism, %(N)	14.3 (12)	15.5 (13)	19.0 (16)	12.0 (10)	
Other determined causes, %(N)	2.4 (2)	2.4 (2)	1.2 (1)	0.0 (0)	
Undetermined causes, %(N)	17.9 (15)	14.3 (12)	11.9 (10)	14.5 (12)	
**Brain MRI findings**					
Left/Right/Both, %	52.4/40.5/7.1	40.5/53.6/6.0	41.7/51.2/7.1	53.0/42.2/4.8	0.624
Cortical/Subcortical only, %	46.4/53.6	39.3/60.7	45.2/54.8	47.0/53.0	0.734
Single/Multiple, %	51.2/48.8	58.3/41.7	53.6/46.4	49.4/50.6	0.680

^a^Pre-stroke cognitive impairment was evaluated with the Informant Questionnaire on Cognitive Decline in the Elderly (IQCODE).

Bold values followed by * are significant at α = 0.05.

*NIHSS* National Institute of Health Stroke Scales, *SD* Standard deviation, *IQR* Interquartile range, *MRI* Magnetic Resonance Image.

**Figure 1:**
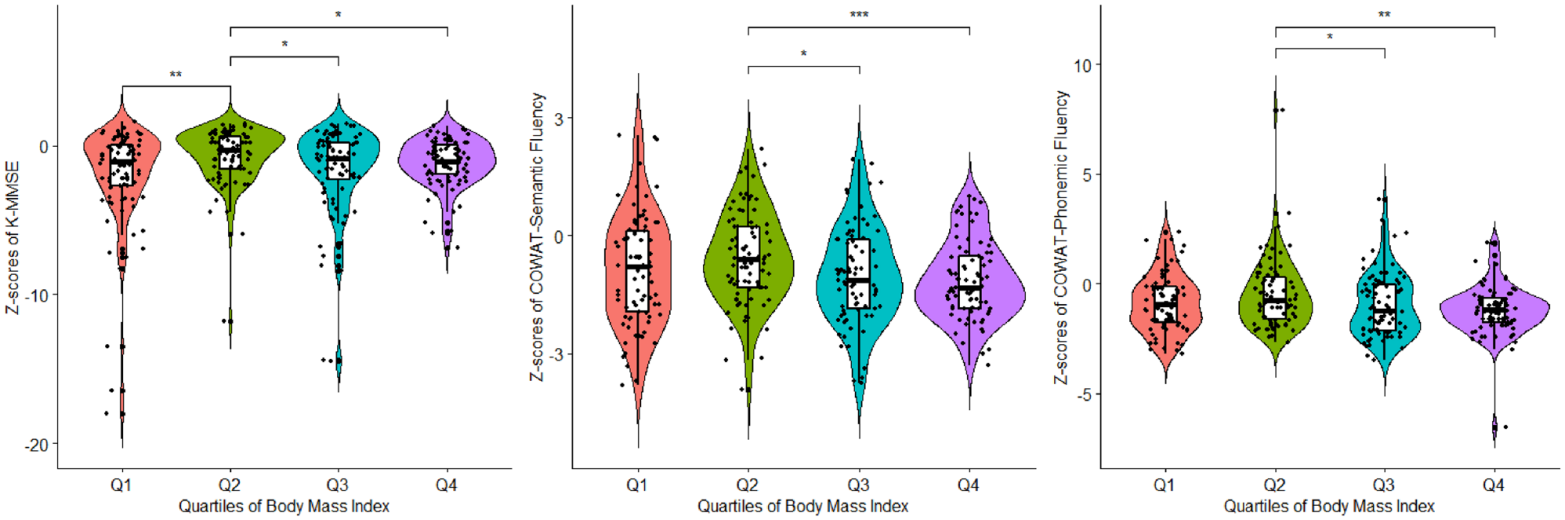
Violin plots indicating the association between the quartiles of BMI and z-scores of both K-MMSE and frontal cognitive domain in patients with ischemic stroke. The z-scores of K-MMSE, COWAT-semantic, and COWAT-phonemic were significantly different between the groups after adjusting for initial stroke severity. (*p* values, 0.014, 0.034 and 0.023 respectively; ANCOVA) *Abbreviations: *BMI*,body mass index; *K-MMSE* Korean version of mini-mental status examination; *COWAT* controlled oral word association test, *ANCOVA* analysis of covariance.

The Z-scores of the K-MMSE differed significantly between the quartile groups at 3 months post-ischemic stroke onset after adjustment for a prespecified covariate, initial stroke severity(NIHSS). (*p* = 0.014, η^2^p = 0.031, ANCOVA, Fig. 1 and Table 2). A multiple comparison analysis indicated that stroke survivors in the Q1 BMI group showed significantly lower global cognitive functions (K-MMSE Z-score, − 2.1 ± 3.4) than those in the Q2 and Q4 BMI groups (K-MMSE Z-score, Q2: − 0.71 ± 1.95; Q4: − 1.21 ± 1.65). The Z-scores of the COWAT also significantly differed between the quartile groups after adjusting for initial stroke severity. A multiple comparison analysis of the COWAT results indicated that participants in the Q4 BMI group had significantly lower phonemic and semantic word fluencies than their counterparts in the Q2 BMI group, despite similar levels of global cognitive function.(*p* = 0.016, η^2^p = 0.032 and *p* = 0.023, η^2^p = 0.029 respectively; ANCOVA). Other neuropsychological test findings relating to frontal lobe functions, including processing speed and mental set-shifting, and other cognitive domains, did not differ significantly between the groups. The sensitivity analysis using Asian-Pacific BMI classification resulted in the loss of statistical significance in comparison of z-scores of K-MMSE and COWAT tests. Only the K-MMSE raw score differences remained significant. (Supplemental Table 2).

**Table 2:** Comparison of Z scores of cognitive tests according to the quartiles of body mass index.

	Q 1 (< 21.87, n = 84)	Q 2 (21.88–23.87, n = 84)	Q 3 (23.88–25.96, n = 84)	Q 4 (> 25.97, n = 83)	Unadjusted *P* values	Adjusted *P* values*
K-MMSE, Z score	− 2.1 ± 3.4	− 0.71 ± 1.95	− 1.68 ± 3.03	− 1.21 ± 1.65	0.007*	0.014*
Raw score	22.99 ± 6.7	25.23 ± 5.21	24.89 ± 5.32	24.56 ± 5.52	0.025*	0.034*
COWAT semantic	− 0.87 ± 1.41	− 0.58 ± 1.18	− 1.03 ± 1.24	− 1.14 ± 1.00	0.019*	0.023*
COWAT phonemic	− 0.88 ± 1.30	− 0.47 ± 1.57	− 0.96 ± 1.44	− 1.18 ± 1.16	0.012*	0.016*
DSC	− 1.06 ± 1.22	− 0.70 ± 1.06	− 1.05 ± 1.37	− 1.11 ± 0.96	0.112	0.159
TMT-A	− 1.44 ± 3.41	− 1.03 ± 2.29	− 1.36 ± 2.84	− 0.62 ± 1.50	0.205	0.315
TMT-B	− 1.13 ± 2.32	− 0.89 ± 1.96	− 1.06 ± 2.09	− 1.09 ± 2.20	0.920	0.954
K-BNT	− 0.95 ± 2.12	− 0.69 ± 1.98	− 1.33 ± 2.76	− 0.67 ± 1.58	0.181	0.236
RCFT Copy	− 1.53 ± 2.44	− 0.88 ± 1.48	− 1.63 ± 2.51	− 1.35 ± 2.06	0.128	0.224
SVLT-E	− 0.83 ± 1.11	− 0.89 ± 1.14	− 1.08 ± 1.29	-0.95 ± 1.19	0.557	0.603

Adjusted for the initial stroke severity (National Institute of Health Stroke Scale).

Bold values followed by * are significant at α = 0.05.

*K-MMSE* Korean Mini-Mental Status Examination; *COWAT* Controlled Oral Word Association Test; *DSC* Digit Symbol Coding; *TMT* Trail Making Test; *K-BNT* Korean version-Boston Naming Test; *RCFT* Rey Complex Figure Test; *SVLT-E* Seoul Verbal Learning Test-Elderly’s version.

## Discussion

In this study, we assessed the association between obesity phenotypes defined using BMI quartiles at admission and cognitive function at 3 months post-ischemic stroke onset. To our knowledge, this study is the first to evaluate BMI and domain-specific cognitive outcomes using a comprehensive, standardized, neuropsychological protocol in relation to a multicenter cohort of stroke patients^24^. Our data analysis indicated that a lower BMI at admission was associated with a higher risk of global cognitive impairment, while a higher BMI was associated with significantly worse frontal dysfunction post-ischemic stroke. These findings suggest that BMI has differential effects on global cognitive function and frontal domain performance following an ischemic stroke.

Previous epidemiologic studies concerning the relationship between BMI and cognitive impairment have yielded controversial findings. The relationship between obesity and long-term cognitive outcomes has alternately been identified as direct, inverse, U-shaped, or even absent^31,32^. However, as previous studies only performed the MMSE to assess cognition, they might have overlooked the influence of BMI on frontal/executive function, given that the MMSE is not sensitive to evaluating frontal lobe function^33^. Our results are in line with previous studies showing that a low BMI was significantly associated with a high risk of cognitive decline^8^.

Despite uncertainly concerning the precise mechanism underlying post-stroke worsening of cognitive function, decreased body weight has been identified as an early indication of declining health and even neurodegeneration^34^. Moreover, several studies have proposed that leptin, an adipokine produced by adipose tissue, exerts neuroprotective effects through anti-oxidative activity and its promotion of hippocampal progenitor cell proliferation. As underweight patients may have decreased levels of leptin, a lower BMI may result in less neuroprotection after neurological insult^35^. It is also possible that low BMI may have enhanced neurodegeneration via nutritional deficiencies including decreased level of brain-derived neurotrophic factor^36^ and vitamin B levels with a reactive elevation of homocysteine^37^.

Our results also showed that a higher BMI was significantly associated with worse frontal/executive function, specifically in phonemic and semantic fluencies. This finding is in agreement with those of previous studies that have linked obesity to temporal atrophy^22^. Moreover, one study that used high-resolution 3D MRI scans reported that obese individuals had a significantly lower density of gray matter in the frontal lobe, post-central gyrus, and middle frontal gyrus than control group participants^23^. Obesity has also been reportedly related to executive dysfunction with other cognitive functions preserved, even in neurologically healthy adults without cognitive impairment^20^. Specifically, obese adults perform worse on executive function tests, especially those testing verbal interference, than their counterparts with a lower BMI. These findings may indicate vulnerability of the frontotemporal lobe in obese patients to acute stroke, regardless of lesion location.

Taken together, lower BMI and higher BMI may have differential pathophysiological roles in cognitive impairment after stroke. These differential effects of BMI on post-stroke cognitive impairment observed in the study may strengthen the clinical utilization of Montreal Cognitive Assessment (MoCA) over MMSE in stroke population^38,39^. The MoCA is known to pick up substantially more cognitive abnormalities after stroke than MMSE by demonstrating deficits in frontal function including verbal fluencies^38^. Our data showed that those with the higher BMI (Q4, BMI > 25.97) had higher K-MMSE scores but lower phonemic and semantic fluency scores compared to the Q1, implying that the stroke population with higher BMI require MoCA or domain-specific neuropsychological battery for optimal screening and diagnosis of cognitive impairment after stroke.

On the other hand, the sensitivity analysis using the Asian-Pacific BMI classification^30^ revealed that statistical significances regarding the differences in z-scores of K-MMSE and each cognitive domain disappear, although both underweight and obese group shows relatively poor cognitive prognosis. The Asian-Pacific classification resulted in only 4% of our patients included in the underweight group (BMA < 18.5). Given this skewed distribution of BMI in study population, it may not be appropriate to assess the risk of post-stroke cognitive impairment according to the Asia–Pacific WHO classification. Our results suggest that if the patients belong to the group with a lower normal range of BMI according to the Asian-Pacific classification^30^, they may be at risk for post-stroke cognitive impairment. These findings are in line with the previous study that having BMI lower than 20 kg/m^2^ was associated with an increased risk of dementia^8^. Those corresponding to our Q2 group (BMI 21.88–23.87 kg/m^2^) are the most advantageous in terms of cognitive prognosis after stroke. This observation should be replicated in future prospective studies and is an important research topic that should be of interest as a potentially modifiable factor.

Our study had some limitations. First, only participants’ height and weight measurements were used to determine the BMI. Other adiposity data, such as abdominal circumference or waist-hip ratio, were unavailable; therefore, we could not address possible differences between leanness and being underweight and their relationships with long-term cognitive outcomes^40^. Future studies should consider replicating the approach of studies that have investigated the relationship between obesity and Alzheimer’s disease through using the waist-to-hip ratio and waist circumference at multiple sites as a measure of central obesity. Second, we were unable to determine any causal relationships between BMI and cognition due to the nature of the cross-sectional study design. Thus, there is room for other interpretations in the association between BMI and differential cognitive outcomes in that they may be due to pre-existing differential cognitive dysfunction. However, the pre-morbid cognitive decline status evaluated with IQCODE was not different between the BMI groups. Third, restrictions were inevitable concerning the number of participants studied and some of the explanatory variables because this study was the result of a secondary analysis of a multicenter study that aimed to investigate the prevalence of cognitive disorders post-stroke. Consequently, we did not include several image variables such as cerebral atrophy or white matter hyperintensities, which may have affected cognitive function post-stroke. Furthermore, we did not assess temporal changes in BMI from stroke onset. Nonetheless, the main strength of our study is that it is by far the first study to investigate the effects of BMI on each cognitive domain after stroke. These findings may provide additional evidence for pathophysiological aspects of BMI on each neural substrate after ischemic stroke.

## Conclusions

Our research suggests that BMI may interact variably with cognitive domains post-ischemic stroke. Although being underweight might negatively affect global cognition post-stroke, obesity might also induce frontal lobe dysfunctions, specifically in terms of phonemic and semantic word fluency. Though exact pathomechanisms being unclear, these findings may be attributed to the distinct roles of BMI on neural substrates, specifically the frontal lobe area. A large prospective cohort study with a focus on neural substrates evaluated with brain imaging is necessary to elucidate further relationships between BMI and post-stroke cognitive outcomes and to validate our findings.

## Supplementary Information

Supplementary Information.

## Data Availability

The datasets generated during and/or analyzed during the current study are available from the corresponding author on reasonable request.
