# Supplementary material for: Differential effects of body mass index on domain-specific cognitive outcomes after stroke
Source: Sci Rep. 2021 Jul 8;11:14168. doi: 10.1038/s41598-021-93714-7 (PMC8266804; doi:10.1038/s41598-021-93714-7)
Supplement: Supplementary file 1 — Supplementary Information. [file 41598_2021_93714_MOESM1_ESM.docx]

**Supplemental Table 1. Korean version of the 60-minute Vascular Cognitive Impairment Harmonization Standards-Neuropsychology Protocol^1^**

| **Cognitive Domains** | **Korean Version of VCIHS-NP protocol** |
| --- | --- |
| Executive/Activation | Animal Naming (semantic fluency) **^2^** |
|  | Korean Controlled Oral Word Association Test (ㄱ,ㅇ,ㅅ) **^2^** |
|  | Digit Symbol-Coding**^3^** |
|  | Korean-Trail making Test-Elderly’s version**^4^** |
| Language | Korean-Boston Naming test: Short form A**^5^** |
| Visuospatial | Rey Complex Figure Test Copy**^6^** |
| Memory | Seoul Verbal Learning Test**^6^** |
| Others | Informant Questionnaire of Cognitive Decline in the Elderly (IQCODE) **^7^** |
|  | Korean-Mini-Mental State Examination**^8^** |
|  | Korean-Instrumental Activity of Daily Living**^9^** |

**References**

1. Yu KH, Cho SJ, Oh MS, Jung S, Lee JH, Shin JH, et al. Cognitive impairment evaluated with vascular cognitive impairment harmonization standards in a multicenter prospective stroke cohort in Korea. Stroke. 2013;44:786-788

2. Kang YW, Chin JH, Na DL, Lee JH, Park JS. A normative study of the Korean version of Controlled Oral Word Association Test (COWAT) in the elderly. Korean J Clin Psychol. 2000;19:385-392

3. Yum TH, Park YS, Oh KJ, Kim JH, Lee YH. Manual for Korean-Wechsler Adult Intelligence Scale. Seoul: Korea Guidance; 1992.

4. Yi H, Chin JH, Lee BH, Kang Y, Na DL. Development and validation of Korean version of trail making test for elderly persons. Dement Neurocognitive Disord. 2007;6:54-66

5. Kang Y, Kim HH, Na DL. A short form of the Korean-Boston Naming Test (K-BNT) for using in dementia patients. Korean J Clin Psych. 1999;18:125-138

6. Kang Y, Na DL. Professional manual; Seoul neuropsychological screening battery. Seoul: Human brain research and consulting; 2003.

7. Lee DW, Lee JY, Ryu SG, Cho SJ, Hong CH, Lee JH, et al. Validity of the Korean version of Informant Questionnaire on Cognitive Decline in the Elderly(IQCODE). J Korean Geriatr Soc. 2005;9:196-204

8. Kang Y. A normative study of the Korean-Mini Mental State Examination(K-MMSE) in the elderly. Korean J Psych. 2006;25:1-12

9. Kang SJ, Choi SH, Lee BH, Kwon JC, Na DL, Han SH, Korean Dementia Research Group. The reliability and validity of the Korean Instrumental Activities of Daily Living (K-IADL). J Korean Neurol Assoc 2002;20:8~14

**Supplemental Table 2. Comparison of Z scores of cognitive tests according to the Asian-Pacific WHO Classification of BMI**

|  | Underweight  (<18.5,  n=15) | Normal  (18.5-22.9, n=122) | Overweight  (23.0-24.9,  n=75) | Obese  (≥25.0,  n=123) | Unadjusted P values | Adjusted P values* |
| --- | --- | --- | --- | --- | --- | --- |
| K-MMSE, Z score | -2.38±2.57 | -1.34±2.99 | -1.12±2.63 | -1.54±2.36 | 0.365 | 0.369 |
| raw score | 19.87±8.6 | 24.20±5.76 | 25.72±4.75 | 24.78±4.97 | **0.002*** | **0.001*** |
| COWAT semantic | -0.88±1.19 | -0.74±1.31 | -0.84±1.22 | -1.09±1.13 | 0.180 | 0.167 |
| COWAT phonemic | -1.33±0.88 | -0.67±1.49 | -0.77±1.42 | -1.08±1.30 | 0.088 | 0.094 |
| DSC | -1.33±1.15 | -0.90±1.16 | -0.84±1.02 | -1.10±1.25 | 0.293 | 0.249 |
| TMT-A | -1.21±1.47 | -1.30±3.02 | -1.14±2.79 | -0.90±2.10 | 0.726 | 0.732 |
| TMT-B | -1.62±2.57 | -1.02±2.05 | -0.75±1.75 | -1.19±2.36 | 0.505 | 0.518 |
| K-BNT | -1.13±1.54 | -0.86±2.16 | -0.95±2.54 | -0.89±1.97 | 0.970 | 0.991 |
| RCFT Copy | -2.07±1.93 | -1.11±2.05 | -1.25±1.91 | -1.56±2.42 | 0.255 | 0.231 |
| SVLT-E | -0.60±1.24 | -0.87±1.07 | -1.03±1.23 | -0.98±1.25 | 0.536 | 0.542 |

Adjusted for the initial stroke severity (National Institute of Health Stroke Scale)

Bold values followed by * are significant at α=0.05

Abbreviations: K-MMSE, Korean Mini-Mental Status Examination; COWAT, Controlled Oral Word Association Test; DSC, Digit Symbol Coding; TMT, Trail Making Test. K-BNT, Korean version-Boston Naming Test; RCFT, Rey Complex Figure Test; SVLT-E, Seoul Verbal Learning Test-Elderly’s version
